# The role of mitochondrial dysfunction and genetics in female infertility and premature ovarian insufficiency

**DOI:** 10.1093/humupd/dmag022

**Published:** 2026-08-19

**Authors:** Brianna L Kline, Alison G Compton, Ann E Frazier, Svenja Pachernegg, Andrew H Sinclair, Katie L Ayers, Elena J Tucker

**Affiliations:** Genomic Medicine, Murdoch Children’s Research Institute, Melbourne, Victoria, Australia; Department of Paediatrics, University of Melbourne, Melbourne, Victoria, Australia; Genomic Medicine, Murdoch Children’s Research Institute, Melbourne, Victoria, Australia; Department of Paediatrics, University of Melbourne, Melbourne, Victoria, Australia; Victorian Clinical Genetics Services, Murdoch Children’s Research Institute, Melbourne, Victoria, Australia; Genomic Medicine, Murdoch Children’s Research Institute, Melbourne, Victoria, Australia; Department of Paediatrics, University of Melbourne, Melbourne, Victoria, Australia; Genomic Medicine, Murdoch Children’s Research Institute, Melbourne, Victoria, Australia; Department of Paediatrics, University of Melbourne, Melbourne, Victoria, Australia; Novo Nordisk Foundation Centre for Stem Cell Medicine, Murdoch Children’s Research Institute, Melbourne, Victoria, Australia; Genomic Medicine, Murdoch Children’s Research Institute, Melbourne, Victoria, Australia; Department of Paediatrics, University of Melbourne, Melbourne, Victoria, Australia; Genomic Medicine, Murdoch Children’s Research Institute, Melbourne, Victoria, Australia; Department of Paediatrics, University of Melbourne, Melbourne, Victoria, Australia; Genomic Medicine, Murdoch Children’s Research Institute, Melbourne, Victoria, Australia; Department of Paediatrics, University of Melbourne, Melbourne, Victoria, Australia

**Keywords:** premature ovarian insufficiency, POI, mitochondria, mitochondrial disease, infertility, genetics

## Abstract

**BACKGROUND:**

Female infertility occurs in ∼37% of infertile couples, while premature ovarian insufficiency (POI) impacts 1–3.7% of women under the age of 40. POI is clinically heterogeneous, with various genetic pathways associated with its pathogenesis. Mitochondrial diseases (MDs) are a broad group of clinically heterogeneous genetic conditions characterized by aberrantly functioning mitochondria. MDs have a disproportionate burden on organs and tissues with increased aerobic/energy demands, such as the heart, skeletal muscles, brain, and ovaries. The role of mitochondria in female fertility and ovarian reserve is increasingly being recognized.

**OBJECTIVE AND RATIONALE:**

A comprehensive understanding of the role of mitochondria in the maintenance of female fertility is pertinent to better understanding female reproductive potential. In a world with increasing demand for assisted reproductive technologies (ART), due to a considerable rate of global infertility, there is a need to better understand the genes and pathways involved in female reproduction. This review summarizes, evaluates, and explores the current knowledge of mitochondria-associated genes and variants that are implicated in POI, including their function and dysfunction in female reproduction.

**SEARCH METHODS:**

We searched articles in the PubMed database, containing the following key words: premature ovarian insufficiency, mitochondria, mitochondrial, premature ovarian failure, genetics, mitochondrial DNA, mtDNA, mitochondrial protein, infertility, premature menopause, mitochondrial donation, assisted reproductive technologies, electron transport chain, oxidative phosphorylation (OXPHOS), mitochondrial disease, oocyte, oogenesis, meiosis, *in vitro* fertilization, mitoribosome, Perrault syndrome, and ovarioleukodystrophy, in the English-language literature until March 2026.

**OUTCOMES:**

Genetic variants that affect mitochondrial genes/proteins can negatively impact ovarian function. Various mitochondrial pathways are associated with female infertility, reflecting the broad sensitivity of ovarian reserve to mitochondrial dysfunction. Mitochondrial dysfunction and infertility can present in isolation or as part of a syndrome. Infertility in women may be the first clinical sign of a MD. Conversely, POI may be an underappreciated symptom of MDs.

**WIDER IMPLICATIONS:**

This review draws attention to the fact that females with MDs should be monitored for POI so it can be detected early for prompt and appropriate therapeutic interventions, such as hormone replacement therapy. This is known to mitigate the risk of comorbidities such as cardiovascular and bone disease and will optimize long-term health outcomes. Of equal importance, our review highlights the potential for girls and women presenting with apparently ‘isolated’ POI to harbour pathogenic variants in MD-associated genes, therefore putting these individuals at risk of developing further clinical manifestations of MDs. We emphasize the need for surveillance in these cases for hearing loss, vision disturbance, cardiomyopathy, muscle weakness and neurodegeneration, depending on the genetic cause. Given that mitochondrial function is essential to female fertility, future therapies for mitochondria-associated infertility could involve mitochondrial supplementation to improve the mitochondrial fraction or mitochondrial donation to optimize the likelihood of reproductive success. Finally, we also discuss the current landscape of biomarkers as potential early diagnostic tools for POI. Whilst currently rudimentary in their clinical utility, the further development of early screening methods will be invaluable for the detection, diagnosis, and early intervention of POI.

**REGISTRATION NUMBER:**

N/A

## Graphical Abstract

**Graphical Abstract:**
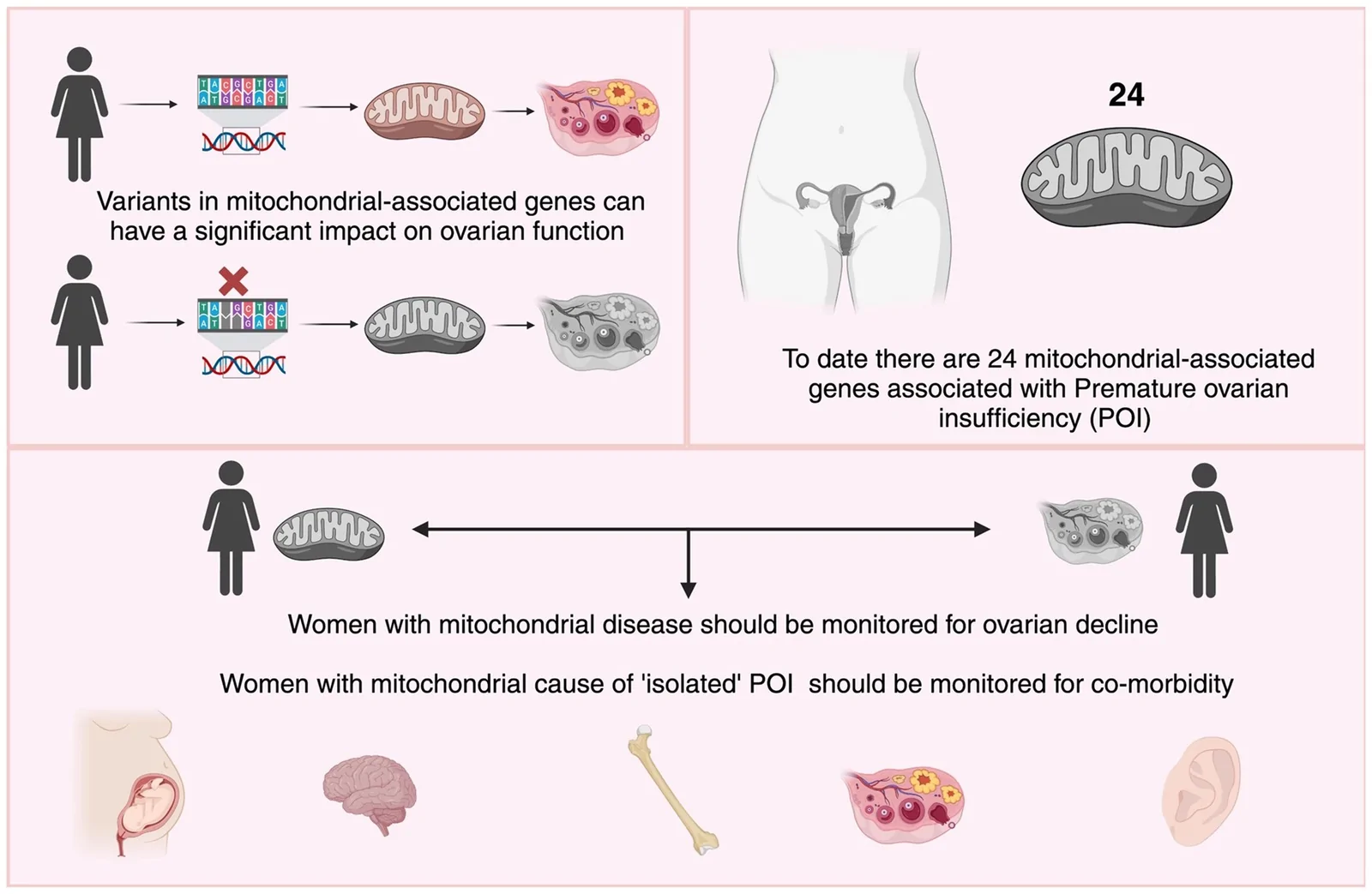
**Mitochondrial function is essential in maintaining fertility potential, since 24 mitochondria-associated genes have been associated, to date, with premature ovarian insufficiency; identifying mitochondrial causes of POI has implications for patient care**. Created in BioRender. Kline, B. (2026). https://BioRender.com/fpmbf4o.

## Introduction

### Infertility: a global health issue

The World Health Organization (WHO) defines infertility as the ‘inability to achieve pregnancy after 12 months or more of regular unprotected sexual intercourse’ (Ombelet, 2020). However, variation in what constitutes ‘infertility’ ranges in medical practice from a lack of pregnancy after anywhere between 12 and 60 months of regular unprotected sexual intercourse (Gurunath *et al.*, 2011), while other definitions exclude a timeframe altogether. Infertility can have significant physiological, psychological, and economic impacts for affected individuals and is believed to be experienced by approximately 1 in 6 reproductive couples or ∼186 million people worldwide (World Health Organization, 2023).

Inconsistent definitions of infertility and data collection methods contribute to varied estimates of global infertility prevalence (Schmidt and Munster, 1995; Boivin *et al.*, 2007; Gurunath *et al.*, 2011; Cox *et al.*, 2022). A meta-analysis of pooled estimates of infertility (between 1990 and 2021, based on a definition of lack of clinical pregnancy after 12 months of regular unprotected sexual intercourse, excluding social infertility) places the global rate at approximately 12.6% (Cox *et al.*, 2022), with female infertility occurring in approximately 37% of infertile couples. Reasons for infertility are heterogeneous, with causes ranging from genetic or environmental causes, iatrogenic causes (such as chemotherapeutic or surgical interventions), advanced maternal age, increased body mass index (BMI), endocrine disorders, infection, immunological conditions, pelvic disorders, and ovarian/uterine/cervical dysfunction and/or anomalies (Shah *et al.*, 2003). However, in approximately 17–26% of couples, infertility may be idiopathic (Garolla *et al.*, 2020; Awonuga *et al.*, 2025).

### Premature ovarian insufficiency

Premature ovarian insufficiency (POI) is characterized by the loss or absence of ovarian activity in women under the age of 40. POI is prevalent worldwide, affecting about 1–3.7% of women (Tucker *et al.*, 2016; Golezar *et al.*, 2019; Heddar *et al.*, 2022). Clinical presentations can include premature loss of menses (secondary amenorrhea) or pubertal failure and primary amenorrhea (complete absence of menstrual periods) associated at times with a complete absence of gonadal structures (streak gonads/gonadal dysgenesis). POI is diagnosed by elevated (>25 mlU/ml) follicle-stimulating hormone (FSH) measured in blood, as per the European Society of Human Reproduction and Embryology (ESHRE) guidelines (De Vos *et al.*, 2010; Sullivan *et al.*, 2016; Eshre *et al.*, 2025).

POI is genetically heterogeneous with >100 genes implicated to date (Touraine *et al.*, 2024). Identification and classification of causative genes and variants is pertinent for clinical management and prevention of comorbidities. Comorbidities of POI include cardiovascular disease, mental health disorders, osteoporosis, and premature mortality (Tucker *et al.*, 2016). Genes identified thus far that contribute to POI include, but are not limited to, genes encoding transcription/translation factors involved in DNA repair/replication, cell differentiation/development, hormone signalling, metabolism and mitochondrial maintenance/structure, mitochondrial DNA replication, and mitochondrial function (Fortuno and Labarta, 2014; Jaillard *et al.*, 2020; Kline *et al.*, 2022).

A growing body of work associates infertility with aberrant mitochondrial function (Demain *et al.*, 2017). Assisted reproductive technologies (ART) have indicated that pregnancy success is linked to oocyte and sperm mitochondrial quality (Subira *et al.*, 2025). Indeed, mitochondrial enrichment therapy or mitochondrial donation may increase reproductive success and should be considered a future therapeutic possibility (Subira *et al.*, 2025). Mouse models have demonstrated dysfunction of mitochondrial proteins, which result in infertility (Lu *et al.*, 2008; Gispert *et al.*, 2013; Cozzolino *et al.*, 2023). Therefore, it is unsurprising that genes and biological pathways affecting mitochondrial function are becoming of increased interest in the field of reproduction.

This review (i) summarizes known POI genes that encode mitochondrial proteins, (ii) explores the biological pathways in mitochondria that are associated with infertility, (iii) highlights biomarkers currently being explored via multi-omic strategies, and (iv) discusses the current landscape of mitochondrial co-factor supplementation: a potential avenue for the treatment of infertility. This review has implications for clinical practice by illustrating the importance of accurate and timely diagnosis of POI associated with mitochondrial dysfunction, especially for surveillance of associated pathologies, and the importance of monitoring ovarian function in women suffering from mild MDs.

## Methods

We searched articles in the PubMed database, containing the following key words; premature ovarian insufficiency, mitochondria, mitochondrial, premature ovarian failure, genetics, mitochondrial DNA, mtDNA, mitochondrial protein, infertility, premature menopause, mitochondrial donation, assisted reproductive technologies, electron transport chain, oxidative phosphorylation (OXPHOS), mitochondrial disease, oocyte, oogenesis, meiosis, *in vitro* fertilization, mitoribosome, Perrault syndrome and ovarioleukodystrophy, in the English-language literature until March 2026.

## Genes encoding mitochondrial proteins associated with premature ovarian insufficiency

The mitochondrial proteome contains >1000 proteins (Rath *et al.*, 2021), most of which are encoded by the nuclear genome. However, the maternally inherited mitochondrial genome (mtDNA) encodes 13 mitochondrial proteins, 2 ribosomal ribonucleic acids (rRNAs), and 22 transfer ribonucleic acids (tRNAs) critical for mitochondrial function. Multiple mitochondrial processes are implicated in reproductive function and ageing. Currently, 24 genes encoding mitochondrial proteins have been identified as contributors to isolated and/or syndromic POI (Fig. 1). These genes are involved in a variety of mitochondrial processes such as mtDNA transcription and translation, mtDNA gene expression, mitochondrial ribosome (mitoribosome) function, mitochondrial protein degradation, and mitochondrial structure and function (Fig. 1).

**Figure 1.**
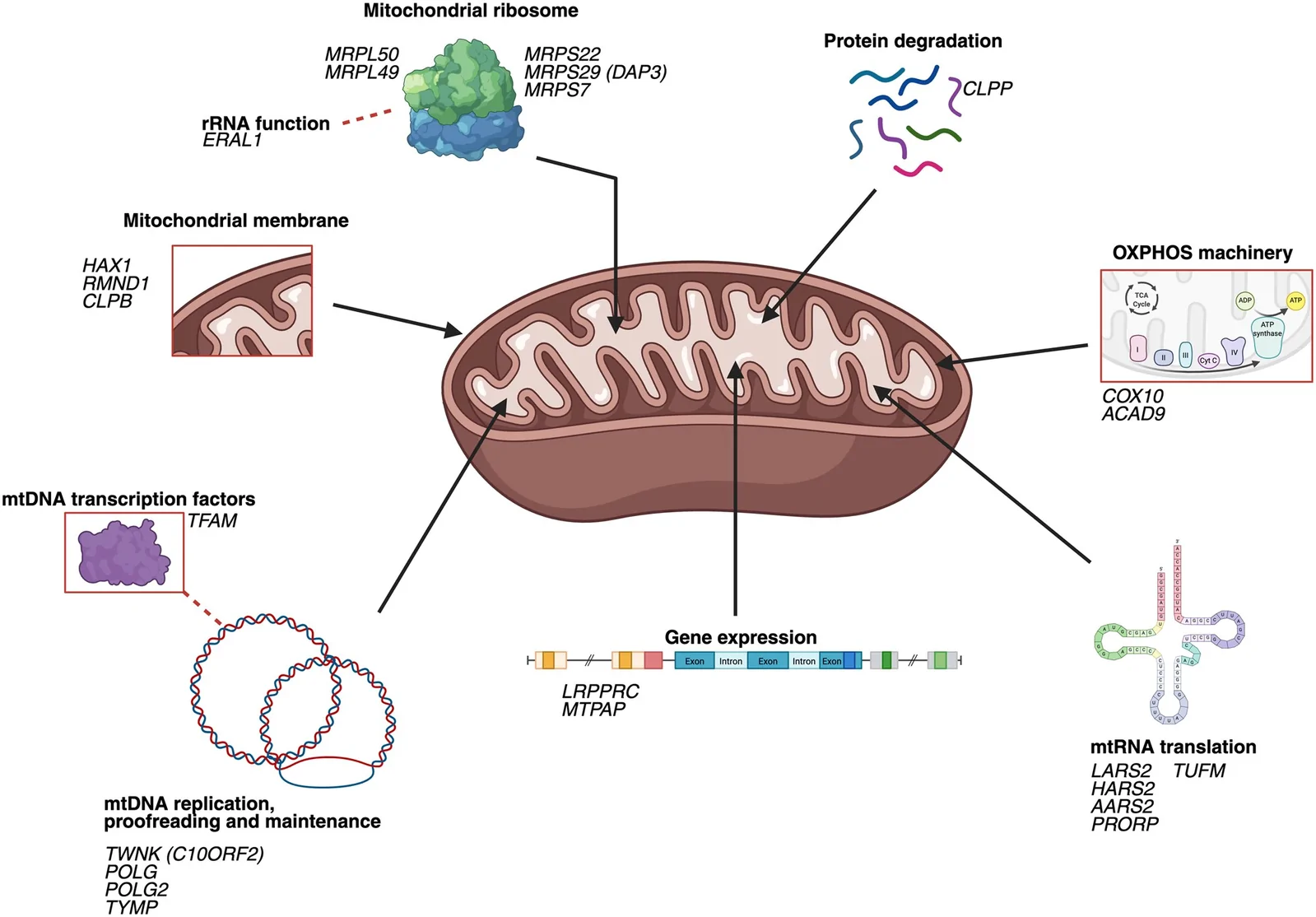
**Mitochondrial genes associated with premature ovarian insufficiency (POI)**. A multitude of mitochondrial pathways/components have been implicated in POI pathogenesis. These processes include protein degradation, OXPHOS machinery, mtRNA translation, gene expression, mtDNA replication, proofreading and maintenance, mtDNA transcription, mitochondrial membrane, rRNA function, and mitochondrial ribosome structure and function. Created in BioRender. Kline, B. (2026) https://BioRender.com/b1jeiuy.

### mtDNA maintenance, replication and transcription in POI pathogenesis

mtDNA replication occurs independently of the nuclear genome, and mtDNA has its own mitochondria-specific replication proteins. These include DNA polymerase γ (*POLG*), the sole DNA polymerase involved in mtDNA replication and associated subunit DNA polymerase γ 2 (*POLG2*), twinkle DNA helicase (*TWNK*), mitochondrial single-stranded DNA binding protein (*SSBP1)*, and mitochondrial RNA polymerase (*POLRMT*/*mtRNAP*) (Wanrooij and Falkenberg, 2010; Holt and Reyes, 2012; Dosekova *et al.*, 2020).

*POLG* variants associated with POI have been identified within the polymerase and catalytic protein domains, often leading to syndromic disease (Luoma *et al.*, 2004; Pagnamenta *et al.*, 2006; Blok *et al.*, 2009; Bekheirnia *et al.*, 2012). Compound heterozygous variants have also been identified in association with POI (Table 1) (Luoma *et al.*, 2004; Naviaux and Nguyen, 2005; Pagnamenta *et al.*, 2006; Bekheirnia *et al.*, 2012; Dosekova *et al.*, 2020; Shen *et al.*, 2021). There has also been one report of syndromic POI associated with the POLG accessory subunit, POLG2 (Dosekova *et al.*, 2020). The clinical presentation of POI associated with *POLG* variants varies, with both primary and secondary amenorrhea observed. In some cases, POI diagnosis precedes other *POLG*-associated disease manifestations such as ataxia and Parkinson’s disease (Van Goethem *et al.*, 2001; Luoma *et al.*, 2004; Winterthun *et al.*, 2005; Persani *et al.*, 2010; Yasuda *et al.*, 2019). This is pertinent, as infertility or aberrant pubertal development may be a primary indicator of subsequent multisystem disease in *POLG* patients.

**Table 1.** mtDNA maintenance, replication and transcription genes associated with premature ovarian insufficiency.

Gene name	Symbol	OMIM	Function	Inheritance	POI associated variants	Other associated disease	References
DNA polymerase γ	*POLG*	174763	mtDNA polymerase	AD	Y955C R943H	Progressive external ophthalmoplegia Parkinsons disease Alpers-Huttenlocher syndrome Ataxia Mitochondrial DNA depletion syndrome 4A/4B Mitochondrial neurogastrointenstinal encephalomyopathy-like syndrome	Luoma *et al.* (2004) Pagnamenta *et al.* (2006) Blok *et al.* (2009) Bekheirnia *et al.* (2012) Shen *et al.* (2021) Van Goethem *et al.* (2001) Naviaux and Nguyen (2005) Winterthun *et al.* (2005) Yasuda *et al.* (2019)
				AR	Y951N N468D A1105T E944D/I941V		
DNA polymerase γ 2	*POLG2*	604983	mtDNA polymerase	AR	D433Y	Ataxia Mitochondrial DNA depletion syndrome Optic atrophy	Dosekova *et al.* (2020)
Twinkle mtDNA Helicase	*TWNK*	606075	mtDNA Helicase	AR	R391H N585S W441G V507I L112Sfs*3 R302W R463Q R323Q N399S	Mitochondrial DNA depletion syndrome 7 Mitochondrial myopathy Axonal myopathy Ataxia Perrault syndrome Ophthalmoplegia Hypergonadotropic hypogonadism Liver failure	Morino *et al.* (2014) Pierce *et al.* (2016) Chang *et al.* (2024) Demain *et al.* (2017) Koskinen *et al.* (1994) Remtulla *et al.* (2019) Nikali *et al.* (2005) Bermejo-Guerrero *et al.* (2021)
Mitochondrial transcription factor A	*TFAM*	600438	mtDNA transcription factor	AR	R232C	Mitochondrial DNA depletion syndrome 15 Liver failure Perrault syndrome Ovarian dysgenesis Breast cancer	Tucker *et al.* (2020) Stiles *et al.* (2016) Ullah *et al.* (2021) Sotgia *et al.* (2012)
Thymidine phosphorylase	*TYMP*		mtDNA nucleotide base maintenance	AR	Q178*	Gastroparesis Osteoporosis Leukodystrophy	Matheou *et al.* (2026)
Mitochondrial poly(A) polymerase	*MTPAP*	613669	mtRNA post-transcriptional modification	AR	L495R	Progressive spastic ataxia, optic atrophy and learning difficulties Encephalopathy Peripheral neuropathy	Epstein *et al.* (2025) Crosby *et al.* (2010) Van Eyck *et al.* (2020) Hiramatsu *et al.* (2022)
Leucine-rich pentatricopeptide repeat containing protein	*LRPPRC*	607544	mtDNA post transcriptional regulation	AR	A354V Intron variant c.469 + 2T > A	Leigh syndrome (French-Canadian) Congenital lactic acidosis Parkinson’s disease Cancer prognosis/progression	Muthaffar *et al.* (2023) Ghaddhab *et al.* (2017) Cui *et al.* (2019) Gaweda-Walerych and Zekanowski (2013)

AD: autosomal dominant. AR: autosomal recessive.

Another gene involved in mtDNA transcription that is implicated in POI is *TWNK* (previously referred to as *PEO1* or *C10orf2*), which encodes a replicative primase-helicase. Diseases associated with *TWNK* (Table 1) can have autosomal dominant or recessive modes of inheritance (Remtulla *et al.*, 2019) and often include syndromic POI. Structural modelling of recessive *TWNK* variants indicates that these variants affect the oligomeric ring structure of the protein that is directly responsible for *TWNK* helicase activity (Li *et al.*, 2025).

Although autosomal recessive TWNK deficiency often results in severe neonatal disease (Remtulla *et al.*, 2019), it is sometimes associated with Perrault syndrome, a milder mitochondrial disorder associated with POI and sensorineural hearing loss (Li *et al.*, 2025). In contrast, autosomal dominant *TWNK* variants have been reported in patients with adult-onset disease, ranging in age from 28 to 86 years (Bermejo-Guerrero *et al.*, 2021). This highlights the broad phenotypic spectrum of *TWNK* variant-associated pathologies (Morino *et al.*, 2014; Pierce *et al*., 2016). Interestingly, *TWNK* haploinsufficiency has also been suggested to correlate with earlier menopause (Shekari *et al.*, 2023).

Direct transcription of mtDNA is performed by mitochondrial transcription factor A (TFAM), a transcription factor considered critical to overall mtDNA maintenance (Larsson *et al.*, 1998). Specifically, TFAM aids in mtDNA distribution during cell division via its role in compaction and distribution of the mtDNA nucleoid (Kasashima and Endo, 2015). Complete knockout of the mouse ortholog *Tfam* is embryonic lethal, with heterozygous depletion resulting in reduced mtDNA copy number (Larsson *et al.*, 1998). MtDNA depletion is known to be associated with reduced fertility (May-Panloup *et al.*, 2005; Wai *et al.*, 2010). Indeed, biallelic variants in *TFAM* have been identified in human patients presenting with POI in the context of Perrault syndrome (Tucker *et al.*, 2020). The involvement of *TFAM* in ovarian dysgenesis has been validated in Zebrafish, whereby *tfam* ablation resulted in mtDNA depletion and ovarian dysgenesis (Ullah *et al.*, 2021). This suggests that mtDNA depletion is the primary mechanism by which *TFAM* variants cause POI, infertility, and disease (Pagnamenta *et al.*, 2006; Wai *et al.*, 2010; Sotgia *et al.*, 2012; May-Panloup *et al.*, 2016; Stiles *et al.*, 2016; Lee *et al.*, 2023).

Decreased mtDNA copy number is known to be associated with reduced reproductive outcomes (Pagnamenta *et al.*, 2006). Mitochondrial DNA depletion syndrome 1 (MTDPS1), also referred to as mitochondrial neurogastrointestinal encephalomyopathy (MNGIE), is a rare autosomal recessive condition characterized by reduced mtDNA copy number and impaired mitochondrial function (Nishino *et al.*, 1999; Filosto *et al.*, 2018). Variants in the enzyme thymidine phosphorylase (TP), encoded by the *TYMP* gene, have been associated with the condition. TP controls the tightly regulated nucleotide pool present in mitochondria via its role in uridine and thymine base catabolism (Nishino *et al.*, 1999). Nucleotide imbalance can, in turn, negatively impact mtDNA replication. Primary hypogonadism is an uncommon presentation of MTDPS1, and to date only one patient with POI has been described (Matheou *et al.*, 2026). However, considering the emerging pattern of POI in MD patients who survive to reproductive age, it is likely that more cases will be observed in the future.

Overall, the involvement of *POLG*, *POLG2*, *TWNK*, *TFAM*, and *TYMP* in cases of POI highlights the importance of mtDNA maintenance, transcription, and abundance in fertility preservation. This is in keeping with the fact that reduced mtDNA content has been reported in women experiencing infertility, with low mtDNA copy number negatively impacting oocyte fertilization (Reynier *et al.*, 2001). Mechanistically, oocytes with low mtDNA copy number and reduced ATP generation do not reach maturity for ovulation. Indeed, oocytes from women with POI have, on average, a three-fold lower mtDNA copy number compared to oocytes from unaffected individuals (May-Panloup *et al.*, 2005; Wai *et al.*, 2010). In some cases, POI is directly related to depleted mtDNA content and, in turn, disrupted mitochondrial biogenesis (Pagnamenta *et al.*, 2006). Reduced mtDNA copy number in oocytes or follicular fluid may act as a biomarker or indicator of POI, although preliminary studies indicate no significant difference in follicular fluid mtDNA copy number in POI patients. However, the caveat is that POI patients often don’t have mature follicles from which samples can be drawn (Zhou *et al.*, 2023b). Other studies have highlighted that low mtDNA in blood may act as a biomarker for natural fecundity (Busnelli *et al.*, 2018; Zhou *et al.*, 2023b). For certainty, further work in this space needs to be performed.

### mtDNA post-transcriptional processes associated with POI

After transcription, mitochondrial ribonucleic acids (mt-RNAs) undergo several critical processing and maturation steps prior to the formation of functional RNAs. Primary mitochondrial transcripts are interspersed with mitochondrial ribosomal RNAs (mt-rRNAs), mitochondrial transfer RNAs (mt-tRNAs), and mitochondrial messenger RNAs (mt-mRNAs). The first step in mt-RNA processing is endonucleolytic excision of mt-tRNAs (Montoya *et al.*, 1981; Bhatta *et al.*, 2021). This is achieved via the action of two critical enzymes, mt-RNase P and Z (Montoya *et al.*, 1981; Rossmanith *et al.*, 1995; Rossmanith, 2012; Bhatta *et al.*, 2021). *PRORP/MRPP3* encodes for mt-RNase P: the enzyme responsible for 5′ cleavage of tRNAs. *PRORP* variants have been observed in patients with syndromic POI in the context of Perrault syndrome (Hochberg *et al.*, 2021). Indeed, analysis of Perrault patient fibroblasts revealed an increase in unprocessed mt-RNA transcripts and a decrease in OXPHOS proteins (Hochberg *et al.*, 2021; Smith *et al.*, 2023).

After cleavage into individual mt-mRNAs, mt-mRNA transcripts are matured at the 3′ termini via the addition of a polyadenylic acid (Poly-A) tail. Poly-A addition is critical for transcript stability and for the completion of some UAA stop codons, which are not endogenously encoded by mtDNA (Nagaike *et al.*, 2005). The enzyme responsible for this process is Mitochondrial Poly(A) Polymerase (*MTPAP)* (Tomecki *et al.*, 2004). Autosomal recessive variants in *MTPAP* were first observed in six patients with early-onset progressive spastic ataxia, optic atrophy, and learning difficulties (Crosby *et al.*, 2010). The phenotypic spectrum of *MTPAP* has since expanded to include lethal encephalopathy and peripheral neuropathy (Van Eyck *et al.*, 2020; Hiramatsu *et al.*, 2022; Epstein *et al.*, 2025). A recent publication, however, described a patient with neurodevelopmental and movement disorder who, at 17, experienced primary amenorrhea and a diagnosis of POI (Table 1). This is in keeping with many MDs, where early reports describe severe multisystem presentation, but over time a phenotypic spectrum emerges with POI as a consistent feature in milder disease.

The importance of stable mt-mRNA transcripts in ovarian function was reinforced upon the identification of POI-associated variants in the Leucine-rich pentatricopeptide repeat-containing (LRPPRC) protein. LRPPRC is involved in the transcriptional regulation of mt-mRNA (Hou *et al.*, 1994). LRPPRC deficiency decreases mt-mRNA stability and polyadenylation and leads to variations in mt-mRNA transcript abundance (Ruzzenente *et al.*, 2012): biological processes that can have significant downstream and multisystem implications, including POI.

Variants in *LRPPRC* are most commonly associated with Parkinson’s disease, cytochrome *c* oxidase (COX) deficiency and Leigh syndrome (French Canadian type, OMIM 256000): a severe early-onset encephalopathy (Gaweda-Walerych and Zekanowski, 2013; Cui *et al.*, 2019; Rahman, 2023). Pre-pubertal mortality is common, so reproductive phenotypes are not usually evident. However, several cases indicate that females with LRPPRC-associated Leigh syndrome, who survive to reproductive age, present with POI, poor pubertal development and rudimentary ovarian/uterine structures (Table 1) (Ghaddhab *et al.*, 2017; Muthaffar *et al.*, 2023). Taken together, the association of gene variants in *PRORP*, *MTPAP*, and *LRPPRC* draws attention to the importance of correct mt-RNA processing for fertility preservation.

### mtRNA translation and its role in POI pathogenesis

The proteins encoded by mtDNA are synthesized within the mitochondria. Mitochondria harbour unique machinery to perform translation independently of nuclear translation (Rossmanith *et al.*, 1995; Richter-Dennerlein *et al.*, 2015). Variants impacting the proficiency of mitochondrial translation across its various stages have contributed to human disease.

Accurate gene translation relies on pairing of tRNAs with their cognate amino acids via esterification reactions, which are catalyzed by aminoacyl-tRNA synthetases (aaRSs) (Antonellis and Green, 2008; Yao and Fox, 2013). In humans, each of the 20 amino acids has its own cognate aaRS. Mitochondrial aaRSs are nuclear-encoded but are translocated to the mitochondria (Diodato *et al.*, 2014) and are critical for translation of mtDNA-encoded proteins. For example, leucyl-tRNA synthetase *2* (*LARS2*) encodes an enzyme that catalyzes the ligation of leucine to its complementary mt-tRNA (Antonellis and Green, 2008; Ye *et al.*, 2015; Liu *et al.*, 2020b), whereas histidyl-tRNA synthetase 2 (*HARS2*) encodes an enzyme that catalyzes the ligation of histidine to its complementary mt-tRNA. Both *LARS2* and *HARS2* variants have been shown to cause Perrault syndrome, in which POI is a feature (Pierce *et al.*, 2011, 2013). The list of *LARS2 and HARS2* variants and pathologies is expanding (Table 2) (Riley *et al.*, 2016; Solda *et al.*, 2016; Kosaki *et al.*, 2018; Yang *et al.*, 2019; Karstensen *et al.*, 2020; Yu *et al.*, 2020; Souissi *et al.*, 2021; Faridi *et al.*, 2022; Lei *et al.*, 2023; Idyahia *et al.*, 2024; Demain *et al*., 2020). Children or young people presenting with hearing loss should consider genetic testing, as this may lead to an early diagnosis of Perrault syndrome, which may allow for fertility preservation (Carminho-Rodrigues *et al.*, 2020). Conversely, individuals who present with POI may consider monitoring for the future development of hearing loss, particularly if POI is attributed to a known Perrault syndrome gene such as *LARS2 or HARS2* (Neyroud *et al.*, 2023). *Alanyl-tRNA synthetase 2 (AARS2)* encodes another mt-tRNA synthetase. However, unlike *LARS2* and *HARS2*, this gene has not been associated with Perrault syndrome but with ovarioleukodystrophy, which is characterized by leukoencephalopathy and POI (Dallabona *et al.*, 2014; Taglia *et al.*, 2018) (Table 2) as well as both severe (early) and late-onset cardiomyopathy (Euro *et al.*, 2015; Nielsen *et al.*, 2020). *AARS2* variants have also been identified in cases of ‘isolated’ ovarian failure, although it is unclear whether these patients will subsequently develop leukoencephalopathy or cardiomyopathy, or have a truly isolated disease (Zhou *et al.*, 2019).

**Table 2.** Mitochondrial translation genes associated with premature ovarian insufficiency.

Gene name	Symbol	OMIM	Function	Inheritance	POI associated variants	Other associated disease	References
Protein only RNase P catalytic subunit	*PRORP*	609947	Mitochondrial endonuclease	AR	A485V	Perrault syndrome Combined oxidative phosphorylation deficiencies Leukoencephalopathy Lactic acidosis	Hochberg *et al.* (2021) Smith *et al.* (2023) Pierce *et al.* (2013) Demain *et al.* (2017) Neyroud *et al.* (2023) Lerat *et al.* (2016) Kosaki *et al.* (2018) Solda *et al.* (2016) Riley *et al.* (2016) Riley *et al.* (2020) Faridi *et al.* (2022) Idyahia *et al.* (2024) Tucker *et al.* (2020) Lei *et al.* (2023) Pierce *et al.* (2011) Karstensen *et al.* (2020) Lerat *et al.* (2016), Yang *et al.* (2019) Demain *et al.* (2020) Souissi *et al.* (2021) Yu *et al.* (2020) Zhou *et al.* (2019) Taglia *et al.* (2018) Euro *et al.* (2015) Zhang *et al.* (2023) Hershkovitz *et al.* (2019) Fluitt *et al.* (2007) Gokalp *et al.* (2024) Fluitt *et al.* (2007), Valente *et al.* (2007) Liu *et al.* (2024a) Zhong *et al.* (2021) Demain *et al.* (2018) Ozieblo *et al.* (2020) Janer *et al.* (2012)
Leucyl-tRNA synthetase 2	*LARS2*	604544	Aminoacyl-tRNA synthetase, leucine	AR	T522N Y577C I360Ffs*15/T629M R453Q/T629M E294K T519M T300M/E638K M117I A430V R228H/D438G A130T/T700I E413K N153H R663W I360T	Perrault syndrome Lactic acidosis Renal disease Sideroblastic anaemia Cardiac abnormalities Neurological features Neuropathy	
Histidyl-tRNA synthetase 2	*HARS2*	600783	Aminoacyl-tRNA synthetase, histidine	AR	V368L L200V L46Q R87C Y337C R480H R87H	Perrault syndrome Hearing loss	
Alanyl-tRNA synthetase 2	*AARS2*	612035	Aminoacyl-tRNA synthetase, alanine	AR	G113R R199C/F131del R199C/M79K R199C/T871Nfs*21	Ovarioleukodystrophy Ataxia Cardiomyopathy Primary pulmonary hypoplasia	
Tu translation elongation factor, mitochondrial	*TUFM*	602389	Mitochondrial GTPase	AR	G175A	Metabolic and isolated cardiomyopathy Lactic acidosis Optic atrophy Encephalopathy/Leukodystrophy Prognostic tumour marker Alzheimer’s disease Combined oxidative phosphorylation deficiencies	
Required for meiotic nuclear division 1 homolog	*RMND1*	614917	Mitochondria translation	AR	N238S G195R/Y273S	Perrault syndrome Hearing loss Kidney disease Renal dysfunction Intellectual disability Mitochondrial translation deficiency White matter degeneration Combined oxidative phosphorylation deficiencies	

Another gene required for mitochondrial translation is mitochondrial Tu translation elongation *factor* (*TUFM*), which encodes a critical GTPase, elongation factor Tu (EFTU). Active EFTU forms a ternary structure with GTP and facilitates the localization of cognate mt-tRNAs to the A-site of mitoribosomes for subsequent translation (Wang *et al.*, 2021; Liu *et al.*, 2024a). EFTU must select the correct aa-tRNA in the ribosome A site to ensure successful polypeptide elongation (Liu *et al.*, 2024a). Variants in EFTU have the potential to cause mitochondrial translation errors that may be detrimental to mitochondrial function and cell survival (Fluitt *et al.*, 2007; Liu *et al.*, 2024a). There have been many human diseases attributed to variants in *TUFM* (Table 2) (Hershkovitz *et al.*, 2019; Zhong *et al.*, 2021). In most reported cases, *TUFM* variants are associated with infantile lethality, so reproductive function remains unknown (Gokalp *et al.*, 2024). There has been one patient reported with a phenotype of isolated POI, who carried a rare homozygous missense variant in *TUFM* (Zhang *et al.*, 2023), with functional studies supporting a deleterious impact. Because this is an isolated case, further studies are required to determine the contribution of *TUFM* variants to isolated or syndromic POI.

The protein called required for meiotic nuclear division 1 homolog (encoded by *RMND1*) supports the translation of mtDNA-encoded proteins by anchoring the mitoribosome to mt-mRNAs for efficient protein synthesis (Janer *et al.*, 2012). Pathogenic variants in *RMND1* impact mitochondrial translation and are associated with a broad phenotypic spectrum (Table 2) (Janer *et al.*, 2012; Demain *et al.*, 2018; Ozieblo *et al.*, 2020). The first reported variants in *RMND1* were associated with severe disease leading to death within the patients’ first two years of life (Janer *et al.*, 2012). Milder manifestations have since been described, including syndromic POI in the form of Perrault syndrome (Demain *et al.*, 2018). It is theorized that different regions of the RMND1 protein may be more sensitive to genetic variation contributing to the varied phenotypic presentations (Broenen *et al.*, 2019).

Mechanistically, mitochondrial translation is crucial for the synthesis of the 13 critical mtDNA-encoded subunits of the OXPHOS system, which is responsible for generating the majority of cellular energy (Konovalova and Tyynismaa, 2013). A byproduct of OXPHOS is the generation of reactive oxygen species (ROS). Therefore, errors in the mitochondrial translation machinery, such as mitochondrial aaRSs or translation elongation factors, have the potential to lead not only to energy deficiency, but also to excess ROS. Oocytes are particularly susceptible to ROS damage due to their need to maintain genomic integrity for decades (Pandey *et al.*, 2024).

### The mitochondrial ribosome and its involvement in POI pathogenesis

To translate mt-mRNA into proteins, mitochondria rely on the mitoribosome. The mitoribosome is a 55S monosome consisting of a 39S large subunit (mt-LSU) and 28S small subunit (mt-SSU). This structure consists of three RNAs (16S mt-LSU rRNA, 12S mt-SSU rRNA, and mt-tRNA^Val^) and 80 proteins, of which 36 are mitochondria-specific (Amunts *et al.*, 2015). Several mitoribosome structural proteins of both the mt-SSU and mt-LSU have been implicated in POI pathogenesis.

To date, three genes encoding mt-SSU subunits have been associated with POI: mitochondrial ribosomal protein S7 (*MRPS7*), mitochondrial ribosomal protein S22 (*MRPS22*), and death-associated protein 3/mitochondrial ribosomal protein S29 (*DAP3/MRPS29*) (Smits *et al.*, 2011; Lazar *et al.*, 2013; Gopisetty and Thangarajan, 2016) (Table 3).

**Table 3.** Mitochondrial ribosome and protein degradation genes and associated variants in patients with premature ovarian insufficiency.

Gene name	Symbol	OMIM	Function	Inheritance	POI associated variants	Other associated disease	References
Mitochondrial ribosome protein S7	*MRPS7*	611974	Mitochondrial ribosome component protein	AR	M184V K125*/R179H	Renal and hepatic failure Lactic acidemia Sensorineural deafness Breast cancer	Menezes *et al.* (2015) Kline *et al.* (2022) Freckmann *et al.* (1997) Sotgia *et al.* (2012) Chen *et al.* (2018) Lazar *et al.* (2013) Smits *et al.* (2011) Smith *et al.* (2025) Jacques *et al.* (2009), Wazir *et al.* (2012) Mariani *et al.* (2001) Davidsson *et al.* (2007) Thomas *et al.* (2025) Bakhshalizadeh *et al.* (2023) Chatzispyrou *et al.* (2017) Shen *et al.* (2021)
Mitochondrial ribosome protein S22	*MRPS22*	605810	Mitochondrial ribosome component protein	AR	R202H R135Q	Breast cancer Non-small cell lung cancer Cornelia de Lange-like syndrome Cardiomyopathy Brain abnormalities	
Death associated protein 3/mitochondrial ribosome protein S29	*DAP3* *MRPS29*	602074	Mitochondrial ribosome component protein	AR	C395Y/UN T123I/UN E392K	Breast and thyroid cancer Glioblastoma Lymphoma Lymphoblastic leukemia Neurological differences Liver failure Lactic acidosis	
Mitochondrial ribosome protein L49	*MRPL49*	606866	Mitochondrial ribosome component protein	AR	H92P R88C A88C/V42Gfs*2	Microcephaly Learning disabilities Retinal dysfunction Kidney/Renal involvement Leukodystrophy	
Mitochondrial ribosome protein L50	*MRPL50*	611854	Mitochondrial ribosome component protein	AR	V112D	Perrault syndrome Kidney dysfunction Cardiac abnormalities	
Era G-Protein-Like 1	*ERAL1*	607435	Mitochondrial ribosome chaperone protein	AR	N236I	Perrault syndrome	

AR: autosomal recessive. AR: autosomal recessive, UN: unknown.

Both isolated and syndromic POI have been observed in patients with variants in mt-SSU genes (Table 3). Although many variants in mitoribosome subunits lead to lethality before reproductive maturity, recent studies have indicated that these genes can also be implicated in milder MDs including syndromic POI. For example, variants in *MRPS7* were initially identified in association with fatal liver and kidney failure but later found to lead to syndromic POI in the form of Perrault syndrome (Freckmann *et al.*, 1997; Menezes *et al.*, 2015; Kline *et al.*, 2022). Variants in *DAP3/MRPS29* have also been described in association with varied phenotypes, including Perrault syndrome (Table 3) (Mariani *et al.*, 2001; Davidsson *et al.*, 2007; Jacques *et al.*, 2009; Wazir *et al.*, 2012; Smith *et al.*, 2025). Fibroblasts from individuals with DAP3/MRPS29 deficiency have demonstrated disruption to other mitoribsome subunits and a related deficiency in OXPHOS complexes I and IV (Smith *et al.*, 2025). This suggests that the mechanism of DAP3/MRPS29 depletion leading to syndromic POI may be the disruption of the mitochondrial ribosome and subsequent OXPHOS subunit deficiency.

In contrast, variants in *MRPS22* have been reported to cause isolated POI (Chen *et al.*, 2018; Jolly *et al.*, 2019). Interestingly, patients with *MRPS22-*associated POI do not always have a measurable OXPHOS deficiency in fibroblasts, suggesting that isolated POI may be due to either mitochondrial dysfunction independent of OXPHOS, a tissue-specific energy deficit, and/or increased sensitivity of ovarian function to subtle mitoribosome dysfunction (Jolly *et al.*, 2019).

The large subunit of the mitochondrial ribosome consists of 52 proteins. There are currently two subunits with variants linked to POI: mitochondrial ribosome protein L49 (*MRPL49*) and mitochondrial ribosome protein L50 (*MRPL50*), both of which are associated with syndromic POI in the form of Perrault syndrome (Bakhshalizadeh *et al.*, 2023; Thomas *et al.*, 2025) (Table 3). Fibroblasts from patients with *MRPL49* or *MRPL50* deficiency had global disruption of the mt-LSU and measurable OXPHOS deficiency, suggesting an energy deficit may be the cause of ovarian failure (Bakhshalizadeh *et al.*, 2023).

These cases highlight the necessity for mitoribosome function in fertility. As such, it is also vital that the mitoribosome is correctly assembled. Era G-protein-Like 1 (*ERAL1*) encodes one such 28S mt-SSU assembly factor, which acts as an RNA chaperone for the 12S mt-SSU rRNA (Ahnn *et al.*, 1986; Dennerlein *et al.*, 2010). Three unrelated patients with variants in *ERAL1* have been reported with Perrault syndrome (Table 3) (Chatzispyrou *et al.*, 2017). The association of POI with *ERAL1* further highlights the criticality of mitoribosome integrity in maintaining female fertility.

### Mitochondrial protein homeostasis in POI

Tight regulation of protein homeostasis within the mitochondrial matrix is critical to mitochondrial function (Luo *et al.*, 2021). This is achieved via several evolutionarily conserved proteases that stringently control the mitochondrial proteome. Damaged or misfolded mitochondrial proteins can negatively impact mitochondrial function (Yuan *et al.*, 2023). Accumulation of defective proteins may increase the abundance of ROS that are not only known to damage oocytes but also to induce apoptotic pathways within granulosa cells (Yuan *et al.*, 2023), both of which can have an impact on ovarian function and fertility.

A protein involved in the degradation, processing, and chaperoning of proteins located in the mitochondrial matrix is the endopeptidase, caseinolytic mitochondrial matrix peptidase proteolytic subunit (CLPP), which forms the proteolytic subunit of the CLpXP complex alongside the AAA ATPase CLPX (Mabanglo and Houry, 2022). Male and female *Clpp*^−/−^ mice are infertile, have profound deafness and shorter life expectancy when compared to control littermates (Gispert *et al.*, 2013). Homozygous missense and loss-of-function *CLPP* variants are observed in human patients with Perrault syndrome, indicating that *CLPP* is associated with syndromic POI (Jenkinson *et al.*, 2013; Ahmed *et al.*, 2015; Dursun *et al.*, 2016; Lerat *et al.*, 2016; Li *et al.*, 2021) (Table 4). A second protein involved in mitochondrial protein homeostasis, caseinolytic peptidase B protein homolog (CLPB), forms another ATP-dependent disaggregase complex found in the mitochondrial intermembrane space that plays a role in disaggregation/solubilization and refolding of protein substrates (Thevarajan *et al.*, 2020). Variants in *CLPB* are frequently associated with childhood disease including 3-methylglutaconic aciduria and severe congenital neutropenia (SCN) (Kanabus *et al.*, 2015; Saunders *et al.*, 2015; Wortmann *et al.*, 2015; Warren *et al.*, 2022). Our group described the first series of patients with *CLPB* deficiency that survived to adulthood (Tucker *et al.*, 2022), and discovered that POI was a consistent long-term feature in affected women. Infertility was also observed in affected men (Table 4).

**Table 4.** Mitochondrial protein homeostasis genes and associated variants observed in a patient with premature ovarian insufficiency.

Gene name	Symbol	OMIM	Function	Inheritance	POI associated variants	Other associated disease	References
Caseinolytic mitochondrial matrix peptidase proteolytic subunit	*CLPP/ClpP*	601119	Protein degradation, ATP-dependent endopeptidase	AR	T145P C147S c.270 + 4A > G I208M I119L/M230L A210T	Perrault syndrome Increased risk of non-small cell lung cancer Muscle weakness/limb spasticity	Ahmed *et al.* (2015) Jenkinson *et al.* (2013), Lerat *et al.* (2016) Dursun *et al.* (2016) Li *et al.* (2021) Yuan *et al.* (2023) Carlsson *et al.* (2013) Cekic *et al.* (2017) Kızılay *et al.* (2025) Zeidler and Welte (2002) Trebinska *et al.* (2010) Tucker *et al.* (2022) Wortmann *et al.* (2015) Kanabus *et al.* (2015) Warren *et al.* (2022) Saunders *et al.* (2015)
Caseinolytic peptidase B	*CLPB*	616254	Mitochondrial disaggregase ATPase	AR	UN/R417* E435_G436delinsDP/G646V R628C/E639K R561Q	Metabolic disorders Neutropenia/Kostmann’s syndrome Acute myeloid leukemia 3-methylglutaconic aciduria Cataracts Epilepsy	
HCLS1-associated protein X1	*HAX1*	605998	Anti-apoptotic factor	AR	E190* W44* R86* E60Dfs*25	Kostmann’s syndrome Neutropenia Epilepsy/developmental delay Breast, lung, skin cancer	

CLPB is required for the stability, solubility, and functional non-aggregated state of the anti-apoptotic protein, HS-1-associated protein X-1 (*HAX1*) (Kasashima *et al.*, 2006). HAX1 has a role in a broad range of cellular processes, from calcium homeostasis and proteostasis to apoptosis, angiogenesis, and translation (Trebinska *et al.*, 2010; Trębińska-Stryjewska *et al.*, 2023). Like *CLPB*, variants in *HAX1* are associated with severe congenital neutropenia (SCN) (Zeidler and Welte, 2002). Early detection of SCN has led to increased patient survival, which has revealed that females with biallelic *HAX1* variants fail to undergo pubertal development and experience POI (Carlsson *et al.*, 2013; Cekic *et al.*, 2017; Kızılay *et al.*, 2025) (Table 4).

### OXPHOS machinery and its role in POI

OXPHOS reactions occur in the inner mitochondrial membrane and involve the transfer of electrons between a series of four complexes (complexes I–IV). This generates a proton gradient that is harnessed by the fifth complex (complex V) to produce ATP. Interruption of this process perturbs ATP generation and generates harmful ROS, which are detrimental to cell survival. Although many genes affecting mitochondrial function have been implicated in POI, there are no reports of pathogenic variants directly affecting OXPHOS subunits that lead to isolated or syndromic POI, although these subunit genes remain candidate genes for POI. This suggests that pathogenic variants in OXPHOS subunits may not be compatible with life/survival to reproductive age or that reproductive phenotypes are overlooked in cases of severe multi-system disease. However, there have been POI patients with variants reported in genes encoding several OXPHOS assembly factors. *ACAD9*, for example, encodes acyl-CoA dehydrogenase family member 9 (ACAD9), an OXPHOS complex I assembly factor (Schiff *et al.*, 2015). Like many mitochondria-associated proteins, ACAD9 deficiency is associated with a broad range of neurological, renal, cardiac, and musculoskeletal manifestations (He *et al.*, 2007; Collet *et al.*, 2016; Repp *et al.*, 2018). Syndromic POI has been reported in one patient (Collet *et al.*, 2016). It is unclear whether POI is a consistent feature of ACAD9 deficiency, with one report of an affected individual being able to successfully carry a pregnancy to term (Jacobi-Polishook *et al.*, 2020). The follow-up of further patients with ACAD9 deficiency will likely clarify the relationship between this mitochondrial disorder and POI.

Similarly, COX10 is an assembly factor for OXPHOS cytochrome *c* oxidase (complex IV), the terminal complex in electron transport (Shoubridge, 2001; Antonicka *et al.*, 2003). It is required for mitochondrial heme A biosynthesis, which is an essential prosthetic group found in CIV, required to transfer electrons from cytochrome *c* to oxygen. Variants in *COX10* often lead to severe disease such as fatal infantile hypertrophic cardiomyopathy, leukodystrophy and Leigh syndrome with renal involvement (Valnot *et al.*, 2000; Antonicka *et al.*, 2003; Haller *et al*., 1989). Two cases of *COX10* variants in POI have been reported. The first case described a patient with biallelic variants and syndromic disease typical of MDs, in addition to POI (Pitceathly *et al.*, 2013). The second case was from a large cohort study and carried a monoallelic variant with no functional validation and remains of uncertain clinical significance (Ke *et al.*, 2023) (Table 5).

**Table 5.** OXPHOS machinery genes, and associated variants, observed in patients with premature ovarian insufficiency.

Gene name	Symbol	OMIM	Function	Inheritance	POI associated variants	Other associated disease	References
Acyl-CoA dehydrogenase family member 9	*ACAD9*	611103	Oxidative phosphorylation—complex i assembly factor	AR	R433Q	Cardiomyopathy, neurological dysfunction, liver dysfunction Developmental delay and intellectual disability, renal dysfunction, muscular weakness, lactic acidosis	Collet *et al.* (2016) He *et al.* (2007) Repp *et al.* (2018) Pitceathly *et al.* (2013) Ke *et al.* (2023) Valnot *et al.* (2000) Antonicka *et al.* (2003) Haller *et al.* (1989)
Cytochrome c oxidase assembly factor COX10	*COX10*	602125	Oxidative phosphorylation	AR	D336V/R339W Y319Lfs*16	Renal involvement, leukodystrophy Leigh syndrome, anaemia, sensorineural hearing loss, infantile hypertrophic cardiomyopathy Lactic acidosis	

AR: autosomal recessive.

Together, these cases suggest that variants causing aberrant or reduced function of OXPHOS pathways are a potential mechanism that can contribute to POI pathogenesis.

## Combating mitochondrial dysfunction in disease and infertility

As discussed, primary MDs are often fatal in early life, multisystem in presentation, and severe. Consequently, most patients do not survive until adulthood to present with a reproductive phenotype, or if they do, reproductive phenotypes are likely underreported as they are deemed less relevant than immediately life-threatening or life-limiting conditions. However, increased access to genomic sequencing for a diverse range of conditions and symptoms has revealed milder MD phenotypes. This has expanded the clinical spectrum to include adult-onset phenotypes, including reproductive disorders. The association of reproductive disorders with MDs is unsurprising given the substantial body of work associating ovarian function with mitochondrial function (Cecchino *et al.*, 2018). Therefore, it is reasonable to assume that the number of MD genes implicated in POI will increase with the more widespread use of genomic sequencing in diagnosis and advancements in patient management that foster survival beyond puberty.

### The importance of fertility surveillance in women with mild-moderate mitochondrial disease

MDs can manifest at any age and can be highly heterogeneous. Endocrine manifestations are not uncommon but are under-reported or under-recognized as being related to mitochondrial dysfunction (Harvey and Barnett, 1992; Ng *et al.*, 2022). Initial research reports focussing on new disease genes are often biased towards more severe clinical presentations, something which holds true for many of the genes discussed in this review.

*TWNK* variants, for example, were initially identified in infantile-onset neurodegenerative conditions (Koskinen *et al.*, 1994; Nikali *et al.*, 2005) prior to the gene being linked to infertility (Morino *et al.*, 2014). Pathogenic *TUFM* variants were originally identified in an infant with lactic acidosis and fatal encephalopathy in 2007 (Valente *et al.*, 2007), however, it was not until 2023 that *TUFM* variants were associated with POI (Zhang *et al.*, 2023). These are just a couple of the many genes that have historically been implicated in severe multisystem disease and are only now being recognized as involved in POI pathology. It is therefore critical to consider reproductive function in individuals diagnosed with mitochondrial disease that are nearing puberty. This allows for better clinical management and fertility preservation to occur prior to reproductive dysfunction.

Likewise, women diagnosed with mitochondrial disease should be considered for clinical surveillance of post-pubertal gonadal function. Affected females can then consider oocyte preservation while fertility potential exists and can receive prompt intervention with hormone therapy when ovarian function declines (Matsuzaki *et al.*, 2002).

### Criticality of timely diagnosis of women with mitochondrial-associated POI

Knowledge of mitochondrial causes of POI has rapidly expanded in recent years from eight known causative genes in 2019 (Tiosano *et al.*, 2019), to 24 at the time of writing this review. For some genes, while variants have been reported in association with syndromic POI in some individuals, others have presented with isolated POI (Riley *et al.*, 2020; Chang *et al.*, 2024). Importantly, POI may be just the first clinical manifestation, and the full clinical phenotype of mitochondrial disease may evolve or be identified later. This was observed in individuals with *MRPL49* variants who were referred to doctors for a diagnosis of Perrault syndrome because of combined sensorineural hearing loss and POI. The patients were later found to have significant white matter abnormalities upon magnetic resonance imaging (MRI) (Thomas *et al.*, 2025). Similarly, some cases of LARS2 deficiency first manifest as POI but evolve into ovarioleukodystrophy with significant neurodegeneration (Riley *et al.*, 2020; Thomas *et al.*, 2025) or Perrault syndrome with later-onset hearing loss (Neyroud *et al.*, 2023). Therefore, in these cases, genetic diagnosis can guide ongoing clinical care, including neurological and audiological surveillance. This approach allows patients to establish a specific and appropriate multidisciplinary clinical team and access treatments that may slow or manage conditions or even help patients prepare for disease progression (Tucker *et al.*, 2016).

## Multi-omic studies for biomarker discovery and early detection of POI

Currently, the only routine biomarkers for POI include elevated levels of follicle-stimulating hormone (FSH) and abnormal additional hormone markers such as luteinizing hormone (LH), estradiol, and anti-Mullerian hormone (AMH), although the efficacy of testing the latter is debated (Anderson and Nelson, 2020; Mao *et al.*, 2026). Translating laboratory research and gene discovery into useful tools for clinicians and patients is pertinent to the future of POI care (Fig. 2). To date, there are several methods in development for detecting or determining risk of POI. These include gene-specific, microRNA, protein, and metabolite biomarkers (Arefnezhad *et al.*, 2024; Liu *et al.*, 2024b; Lu *et al.*, 2024). However, none have been built into routine care, and therefore require further work to determine their applicability and feasibility.

**Figure 2.**
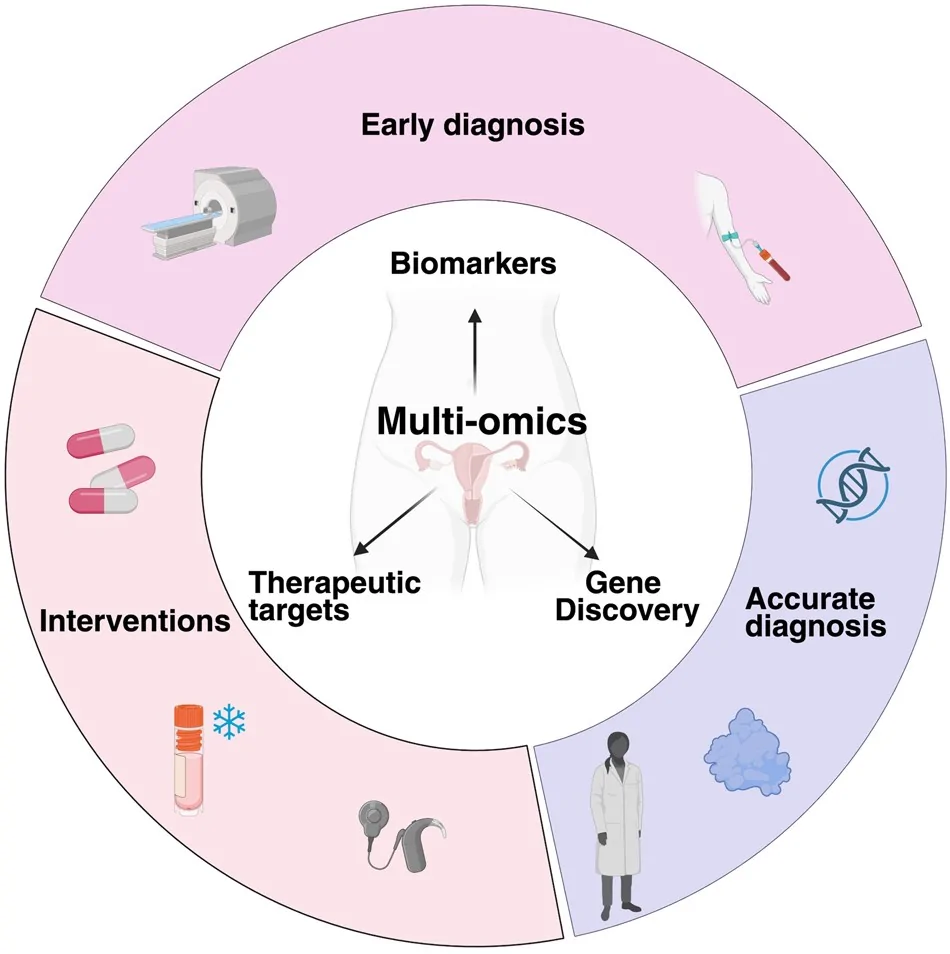
**Multi-omic studies are driving improvements in POI patient care**. Multi-omic research is providing rich resources for improving POI patient care, by enabling (i) earlier diagnosis, made possible by biomarker discovery, that can complement imaging and hormonal studies, (ii) interventions, made possible by the identification of therapeutic targets, such as medications to relieve symptoms or improve fertility, and (iii) accurate diagnosis, made possible by gene discovery, allowing clinicians to understand the underlying pathology and provide accurate counselling and appropriate therapeutic plans for patients. When applied holistically, this approach will help to improve fertility and health outcomes. Image created in BioRender. Kline, B. (2026) https://BioRender.com/fxwfojd.

Improvements in multi-omic approaches allow for the rapid analysis of biomarkers in large POI cohorts. A recent transcriptomic study identified 119 mitochondrial differentially expressed genes (mitoDEGs) in POI patients (Lu *et al.*, 2024), indicating a unique signature of mitochondrial disruption in affected individuals. Of particular interest was the downregulation of mitoribosome genes *MRPL12*, *MRPS7*, and *MRPL51* in human POI patient granulosa cells (Lu *et al.*, 2024). Given the mitoribosome genes identified in monogenic POI to date, these findings hint at general mitoribosome disruption potentially predisposing to ovarian decline. Interestingly, the expression of MitoDEGs was closely associated with immune cell infiltration, indicating the possibility of mitochondrial damage triggering an immune response. Future therapeutic strategies for infertility may, therefore, target mitochondrial integrity and/or the immune response to its disruption. Although a signature indicating mitochondrial disruption was observed in POI women, transcriptomics is usually not offered clinically due to its cost and complexity, and because health care providers are generally not trained to interpret this large-scale data, limiting its clinical utility. Nonetheless, as multi-omic expertize develops globally, transcriptomic-based diagnostics may emerge.

MicroRNAs (miRNAs) are the most abundant class of small RNAs in the ovary and are critical to its function (Otsuka *et al.*, 2008; Baley and Li, 2012) via gene regulation, apoptosis, and cell proliferation. miRNAs have been asserted as contributory to reproductive diseases, such as POI (Arefnezhad *et al.*, 2024), with three miRNA species associated with human POI pathogenesis (Gao *et al.*, 2025) to date. miR-379-5p was differentially expressed in POI patients and found to negatively impact granulosa cell proliferation and DNA repair (Dang *et al.*, 2018). miR-181a is similarly involved in granulosa cell apoptosis and found to be elevated in POI patients (Zhang *et al.*, 2019). Differential miRNA expression has also been observed in autoimmune-associated POI, with miR-21 downregulation observed in patients (Li *et al.*, 2020). Interestingly, miR-21 overexpression improves ovarian function in chemotherapy-treated rats (Fu *et al.*, 2017). Whilst this area of research is interesting, differing and variable miRNA species and ethnicity-specific trends within discovery cohorts mean that further broad-scale studies are required (Wu *et al.*, 2026).

Metabolomics and proteomics can link identified variants with downstream protein abundance and biological pathways that may explain a clinical phenotype. Rapid scientific and technological advancements now allow for the better detection of protein and metabolomic markers in patient serum samples, which are more accessible than ovarian tissues and samples. Combining proteomic and metabolomic profiles of patients may give clinicians greater insights into disease risk and progression. This will be invaluable to clinical care and the establishment of therapeutic targets. For instance, metabolomic and proteomic analysis of extracellular vesicles obtained from serum (14 patients with POI and 16 healthy controls) revealed metabolic signatures that distinguished two subgroups within the POI cohort, one with greater variance from healthy controls and with significant disruption to pyruvate metabolism, glycolysis, and glucagon signalling (Liu *et al.*, 2024b). Likewise, the proteomic analysis suggested that inflammation may be an early factor contributing to POI development. This is reminiscent of the transcriptomic studies that identified energy metabolism and immune regulation as disrupted in POI.

Specifically, Li *et al.* (2024) elucidated that afamin, alpha-1-B glycoprotein (A1BG), 2-oxoarginine, quinolinic acid, and L-phenylalanine were consistently differentially expressed in the POI cohort. However, further research is necessary to establish diagnostic value, due to the small sample size and lack of replication with an independent cohort. Several studies have proposed afamin as relevant to POI, with the protein known to have a protective role against oxidative stress in granulosa cells (Liu *et al.*, 2020a). However, afamin is also a marker for several other diseases such a Lupus (Lin *et al.*, 2025), so the authors proposed its use in conjunction with 2-oxoarginine as a combined biomarker for POI.

Yu *et al.* (2023) conducted a cohort study using untargeted metabolomics in 40 individuals (including 20 who were diagnosed with clinical POI and 20 controls), and found that a 2.1-fold increase in plasma ceramide was associated with reduced plasma Coenzyme Q10 (CoQ10/Ubiquinone) and retinol levels (Yu *et al.*, 2023), therefore highlighting their potential utility as biomarkers for POI (Wu *et al.*, 2026). In a separate metabolomic study of plasma from 30 POI patients and 30 age-matched controls, six metabolites (Arachidonoyl amide, 3-3OH3MB, dihexyl nonanedioate, 18-HETE, cystine, and phosphatidylglycerol PG (16:0/18:1)) were identified to correlate with ovarian reserve and may form a promising combination of biomarkers. Increases in arachidonoyl amides, which are signalling lipids, may contribute to pathogenesis of POI due to their role in the endocannabinoid system, which plays a role in folliculogenesis and ovulation (Zhou *et al.*, 2023a). Blocking or downregulating endocannabinoids in POI patients poses a potential therapeutic target to improve clinical symptoms and comorbidities, such as cardiovascular disease. However, further work is required given the differing metabolite markers identified in different studies (Chen *et al.*, 2021; Zhou *et al.*, 2023a).

Multi-omic research is uniquely positioned to bridge the current gap between gene discovery and treatment. Assessing more broadly downstream biological interactions and functional targets may highlight treatment options that will lead to better outcomes for patients in the future.

## Current mitochondrial therapies for combating infertility

### Mitochondrial co-factor supplementation

Models of reproductive ageing have demonstrated that oocyte quality correlates with nicotinamide adenine dinucleotide (NAD^+^) levels, with NAD + levels declining in oocytes and granulosa cells in ageing animal models (Xie *et al.*, 2020; Liang *et al.*, 2023). NAD^+^ is a metabolic cofactor critical to the mitochondrial OXPHOS system and energy production. Whilst relatively novel as a treatment target for age-related disease, NAD^+^ was first identified over 100 years ago (Berger *et al.*, 2004; Elhassan *et al.*, 2017). Now, NAD + decline is known broadly to be associated with many age-related conditions such as Alzheimer’s disease, metabolic disorders, and cardiac disease (Abdellatif *et al.*, 2021; Hikosaka *et al.*, 2021).

NAD + is believed to lower ovarian inflammation, improve oocyte quality and increase fertility specifically via the inhibition of inflammatory responses to ROS and DNA damage (Liang *et al.*, 2023; Smits *et al.*, 2023). NAD^+^ is known to improve mitochondrial function via the reduction of ROS, improve meiotic spindle assembly, and increase mitochondrial membrane potential. To date, the supplementation of nicotinamide mononucleotide (NMN), which is converted to NAD^+^, has been shown to restore oocyte quality in several animal models (Bertoldo *et al.*, 2020; Yang *et al.*, 2020; Ma *et al.*, 2024; Ramírez-Martín *et al.*, 2025). Yet, whilst NMN supplementation improved NAD + levels and reduced ROS in a mouse model with drug-induced POI, no significant improvement in fertilization or blastocyst formation was observed (Ramírez-Martín *et al.*, 2025). A rat model of POI, generated similarly via chemotherapeutic treatment, demonstrated that NMN supplementation improved hormone levels back to that of the control group (Ma *et al.*, 2024). Ovarian primordial follicle count was improved in the treated POI model but was still reduced when compared to the control (Ma *et al.*, 2024). Regardless, both animal models indicate a potential therapeutic benefit of NMN/NAD^+^ supplementation in improving ovarian function. Although studies illustrate the potential utility of NAD^+^ supplementation in animal models, it is unclear if POI patients with genetic variants impacting mitochondrial function would benefit similarly with this therapy. Nevertheless, this avenue provides a potential therapeutic option for affected individuals, and clinical trials in human patients would be invaluable (Cordone *et al.*, 2026).

Another avenue that may be beneficial to fertility is exogenous supplementation of mitochondrial co-factors. CoQ10/Ubiquinone is a vital component of the OXPHOS process, with a role in transferring electrons from complex I and II to complex III and acting as an antioxidant to protect cells from oxidative damage (Crane *et al.*, 1957; Crane, 2001). It has been proposed that adjuvant oral supplementation of CoQ10 could improve fertility in women aged 31 and older (Akarsu *et al.*, 2017; Giannubilo *et al.*, 2018). In mice, both oocyte number and mitochondrial activity were improved by CoQ10 supplementation (Bentov *et al.*, 2010). CoQ10 supplementation was also shown to preserve the ovarian follicle pool in aged mice (Ben-Meir *et al.*, 2015). Whilst studies of CoQ10 supplementation in infertile patients are promising (Florou *et al.*, 2020; Showell *et al.*, 2020), further studies are required to consolidate the potential of CoQ10 supplementation as a fertility preservation treatment in individuals with POI.

Melatonin, in addition to being a critical hormone regulator of circadian cycles, also acts as a free radical scavenger and can have antioxidant effects (Poeggeler *et al.*, 1993). Melatonin also acts to stabilize the inner mitochondrial membrane, and studies in rats have indicated that melatonin supplementation is protective against mitochondrial oxidative damage (Martin *et al.*, 2000). Studies investigating the impact of oral melatonin on fertilization and oocyte retrieval are conflicting. One study of women undergoing *in vitro* fertilization (IVF) demonstrated that melatonin improved the quantity of oocytes retrieved, fertilization success and embryo quality (Sadeghpour *et al.*, 2025). Melatonin has also been shown to protect against granulosa cell autophagy in ovarian-like cell lines and granulosa cells derived from POI patients (Wu *et al.*, 2022; Li *et al.*, 2024). On the contrary, another study observed that whilst melatonin did increase the percentage of mature oocytes retrieved and subsequent fertilization rate, the data failed to reach statistical significance (Batioglu *et al.*, 2012). Although there is promising theoretical and experimental evidence on the utility of melatonin in POI, further randomized controlled trials are required before such treatment can be adopted into routine care.

The natural polyphenol resveratrol, found in grape skin, red wine and nuts, berries etc., much like melatonin, exerts an antioxidant effect in the mitochondria. Resveratrol does this by activating sirtuin 1 (SIRT1), an NAD+-dependent histone acetylase (Howitz *et al.*, 2003; Lagouge *et al.*, 2006). The compound has also long been thought to possess anti-inflammatory, cardiovascular protective, and anti-cancer benefits (Fremont, 2000). However, the dosage and timing of delivery are critical, as studies have shown that the compound can also interfere with the development of a suitable uterine environment for embryo implantation (Ochiai *et al.*, 2019). Resveratrol has been shown to reduce oxidative stress and inhibit apoptosis in granulosa cells in a rat model of POI (Li and Liu, 2018). Similarly, in POI mouse models, resveratrol suppressed POI progression (Wu *et al.*, 2020; Hu *et al.*, 2024). Human clinical trials have found that resveratrol may improve mitochondrial biogenesis and reproductive function in women with PCOS (Ardehjani *et al.*, 2024). Investigations into resveratrol usage to treat female infertility in humans remains ongoing, with some conflicting outcomes of clinical trials (Conforti *et al.*, 2024).

Importantly, studies of potential therapeutics for POI have not stratified patients based on genetic cause. This may confound results and explain some discrepancies in therapeutic impact in the literature. It is conceivable that therapies that target mitochondrial function will have greatest impact on POI that results from disrupted mitochondrial biology and not that arising from an alternative pathology affecting hormone regulation or ovarian development. It is therefore important that future research investigates the impact of therapeutics on genetically defined cohorts of patients or animals.

### Current whole mitochondrial supplementation therapies in combating infertility

Mitochondrial enrichment has been theorized as a potential therapeutic approach for infertile individuals. It is plausible that cell-free mitochondrial donation for subfertile women could improve reproductive success. This is commonly referred to as mitochondrial replacement therapy (MRT). MRT can be autologous, i.e. using self-mitochondria to improve oocyte quality, or heterologous, i.e. used to prevent the inheritance of pathogenic mtDNA variants (Subira *et al.*, 2025); however the latter is outside the scope of this review. Cell-free mitochondrial therapy involves direct injection of healthy cell-free mitochondria, or mitochondria contained within extracellular vesicles, into a patient’s oocytes. The mitochondria for this approach are derived, ideally, from a patient’s ovarian germline, granulosa cells and/or ovarian stem cells or oocyte precursor cells.

Mouse models have demonstrated that injections of autologous mitochondria can rescue oocyte quality and fertility in aged mice (Wang *et al.*, 2017). This is referred to as ‘oocyte rejuvenation’ and has been considered a possible intervention in human ART. One approach trialled in human patients is autologous germline mitochondrial energy transfer (AUGMENT), in which mitochondria are isolated from laparoscopically obtained oogonia stem cells of a patient (Woods and Tilly, 2015; Subira *et al.*, 2025). The mitochondrial suspension is subsequently injected into oocytes during an intracytoplasmic sperm injection (ICSI) procedure. The AUGMENT method did not improve fertility rates in a study group of women ≤42 years (Labarta *et al.*, 2019). However, a more recent study showed improved fertility in women using AUGMENT, with clinical pregnancy rates increasing from 0% to 27.4% of embryos transferred (Morimoto *et al.*, 2023), reflecting the conflicting reports of treatment efficacy. Again, this could reflect the heterogeneity of POI pathogenesis, with the approach only being beneficial for individuals with mitochondrial-associated POI, highlighting the importance of genetic diagnosis and patient stratification for such studies.

## Conclusion

It is irrefutable that the mitochondria functions as the ‘powerhouse’ of the cell. It is therefore unsurprising, given the energic demands of oocytes, that mitochondrial function is associated with reproductive potential and success. POI is known to have a multitude of genetic causes. Genomic advances have elucidated many genes involved in mitochondrial structure and function, that are also implicated in POI pathogenesis. This review highlights the importance of the mitochondria in female fertility, and details the genes and pathways implicated in POI pathology, to date. Furthermore, this review highlights the need to recognize POI as a potential feature of mitochondrial diseases when individuals survive beyond puberty. This review contends that gonadal and hormonal function should be investigated in post-pubertal patients with mitochondrial disease, allowing for prompt instigation of hormone therapy which can improve long term health outcomes, and the potential for fertility preservation before oocyte depletion, where appropriate. It also highlights the potential for POI to be among the first clinical manifestations of mitochondrial disorders. The identification of a mitochondrial basis for POI has broad health implications and can guide optimal patient care via the assembly of a multidisciplinary care team and surveillance for early detection of other symptoms, which can ultimately improve adjustments and outcomes. Furthermore, a number of mitochondrial-focused therapeutics have shown promise in mouse models or POI cohorts and may be suitable for treating POI due to mitochondrial dysfunction, although further work is required to consolidate these research findings.

## Data Availability

No new data were generated or analyzed in support of this research.
